# Multi-Sensor Fusion with Interacting Multiple Model Filter for Improved Aircraft Position Accuracy

**DOI:** 10.3390/s130404122

**Published:** 2013-03-27

**Authors:** Taehwan Cho, Changho Lee, Sangbang Choi

**Affiliations:** Department of Electronics Engineering, Inha University, Incheon city 402-751, Korea; E-Mails: burujo@naver.com (T.C.); lchh0902@gmail.com (C.L.)

**Keywords:** communications, navigation and surveillance/air traffic management (CNS/ATM), ground-based augmentation system (GBAS), automatic dependent surveillance-broadcast (ADS-B), wide-area multilateration (WAM), interacting multiple model (IMM) filter

## Abstract

The International Civil Aviation Organization (ICAO) has decided to adopt Communications, Navigation, and Surveillance/Air Traffic Management (CNS/ATM) as the 21st century standard for navigation. Accordingly, ICAO members have provided an impetus to develop related technology and build sufficient infrastructure. For aviation surveillance with CNS/ATM, Ground-Based Augmentation System (GBAS), Automatic Dependent Surveillance-Broadcast (ADS-B), multilateration (MLAT) and wide-area multilateration (WAM) systems are being established. These sensors can track aircraft positions more accurately than existing radar and can compensate for the blind spots in aircraft surveillance. In this paper, we applied a novel sensor fusion method with Interacting Multiple Model (IMM) filter to GBAS, ADS-B, MLAT, and WAM data in order to improve the reliability of the aircraft position. Results of performance analysis show that the position accuracy is improved by the proposed sensor fusion method with the IMM filter.

## 1. Introduction

Countries with advanced aviation technologies such as the U.S. and the European nations have shown consistent dedication to improvement of aviation safety for increased air traffic volumes. In addition, research on navigation systems that employ satellite technology has been actively promoted in accordance with the need to improve existing navigation systems. Accordingly, the International Civil Aviation Organization (ICAO) set up the Future Air Navigation System (FANS) and has developed Communications, Navigation, and Surveillance/Air Traffic Management (CNS/ATM)—a new scheme for air traffic management. CNS/ATM incorporates communications, navigation, and surveillance systems that employ digital technologies, including satellite systems, together with various levels of automation in support of a seamless global air traffic management system [1].

The transition to CNS/ATM will have a considerable influence on not only technical aspects of air traffic management such as increasing airspace capacity and providing efficient routes, but also the economics of relevant fields. Thus, ICAO member countries have been committed to technology development and related infrastructural improvement through making global, regional, and national plans. The transition to CNS/ATM is recognized as a revolutionary paradigm shift in air traffic control and an effective tool for air traffic safety, efficiency, and economy. Surveillance in CNS/ATM can be classified into dependent surveillance and independent surveillance. Dependent surveillance is where a pilot monitors aircraft positions on the basis of information conveyed by voice, whereas independent surveillance is where remote aircraft surveillance is performed using primary and secondary radar.

The Ground-Based Augmentation System (GBAS) is designed to enhance the Global Navigation Satellite System (GNSS) to meet the requirements of CNS/ATM. GBAS provides advanced approach, landing and departure services [2]. In Automatic Dependent Surveillance-Broadcast (ADS-B), aircraft broadcast their location and other details such as climbing speed to adjacent aircraft or ground stations for accurate surveillance [3]. Multilateration (MLAT) is employed when the installation and operation of radar is difficult or there is an aircraft surveillance dead zone. MLAT is very useful when such dead zones exist, or when the cost of installing additional radar facilities is prohibitively high because of the structural propagation characteristics of current radar through the airport and major airways. It also can be useful in aircraft detection below a certain altitude and screen jamming due to mountains and obstacles [4]. The basic concept of wide-area multilateration (WAM) is the same as that of MLAT, but it can monitor areas far from the airport. In other words, MLAT monitors aircraft and moving objects in close range such as the airport grounds, and WAM is a system that monitors aircraft on major airways [5]. GBAS, ADS-B, MLAT, and WAM are all next-generation technologies with higher accuracy than existing technologies. However, this paper proposes the use of sensor fusion method with Interacting Multiple Model (IMM) filter for greater reliability.

This paper is organized as follows: Section 2 discusses related work on background, GBAS, ADS-B, MLAT, WAM, IMM filter, and sensor fusion. Section 3 introduces the proposed sensor fusion method applied to the IMM filter. Section 4 presents the performance analysis results for the proposed method. Finally, Section 5 discusses the research implications and presents further research questions.

## 2. Related Works

### 2.1. Background

There have been a lot of discussions and study in the sensor fusion field and sensor fusion methods are widely used in this field. To identify the accurate position of an aircraft, the technique that fuses ADS-B and MLAT/WAM to a rotating sensor was proposed in [6]. Multi-sensor data fusion architectures and techniques were proposed in [7]. Implementation of a multi-sensor tracking algorithm for large scale air traffic surveillance based on IMM state estimation combined with a 2-dimensional assignment for data association was proposed in [8]. In [9], a practical introduction about data fusion methods was provided. In [10], the authors discuss the design and implementation of an algorithm for track formation and maintenance in a multi-sensor air traffic surveillance scenario. An algorithm for multi-platform, multi-sensor fusion with adaptive-rate data communication was presented in [11]. A data fusion architecture for air traffic control applications based on the radar plot and ADS-B was proposed in [12]. MLAT can be used for airport surface movement surveillance as well as terminal and en route surveillance using WAM according to [13]. In [14], the regularization method was proposed to solve the location problem of MLAT by mode S. Various applications of mode S for air traffic and airport traffic management were explored in [15], and multi-sensor data fusion was even used to help reduce sensor failure risk in [16].

### 2.2. GBAS

GBAS is designed to enhance GNSS systems to meet the requirements of CNS/ATM. GBAS augments GNSS to obtain accurate aircraft position and improve aircraft safety. GBAS also supports all phases of approach, landing and departure.

### 2.3. ADS-B

At the center of the CNS/ATM system is the ADS-B system, which is based on digital communications. Traditional surveillance methods include voice reporting, visual checks, and primary and secondary surveillance radars. However, in CNS/ATM, these methods are replaced by ADS-B, a radically new technology that is redefining the paradigm of communications, navigation, and surveillance in air traffic management today. Already proven and certified as a viable low-cost replacement for conventional radar, ADS-B allows pilots and air traffic controllers to see and control aircraft with more precision over a far larger percentage of the Earth's surface than has ever been possible before. ADS-B is a next-generation air surveillance system that will supplant and complement conventional radar, since conventional air traffic management radar systems will reach their limits soon owing to the increases in air traffic. According to recent studies, the position accuracy of conventional radar is 200 m. However, ADS-B achieves a position accuracy of 33 m [17]. Nevertheless, although ADS-B has better position accuracy, it includes errors from the GNSS since the position information of the aircraft relies completely on the GNSS [18].

### 2.4. MLAT

The basic principle of MLAT is the use of a hyperbolic curve and determination of hyperbolic position. These measurements use the time difference of arrival (TDOA) [19] of the received signal from four receivers. One of the receivers is used as a reference, and the remaining three calculate the position of the aircraft. The specific methods for TDOA are as follows [20,21]. The distance between each receiver and the target aircraft is calculated as follows:

(1) $$D_{i}=\sqrt{{\left(x_{i}-x\right)}^{2}+{\left(y_{i}-y\right)}^{2}+{\left(z_{i}-z\right)}^{2}}$$

where (*x_i_*, *y_i_*, *z_i_*) denotes the location of each receiver; i is the number of receivers; and (*x*, *y*, *z*) is the position of the aircraft. In other words, the distance between the aircraft and the receiver can be calculated. If a particular receiver is set as the origin, the distance between that receiver and the aircraft is:

(2) $$D_{0}=\sqrt{x^{2}+y^{2}+z^{2}}$$

If the propagation speed is *v* and propagation travel time is *t*, the difference between Equations (1) and (2) is the product of speed and distance, that is:

(3) $$D_{i}-D_{0}=vt$$

**Figure 1.**
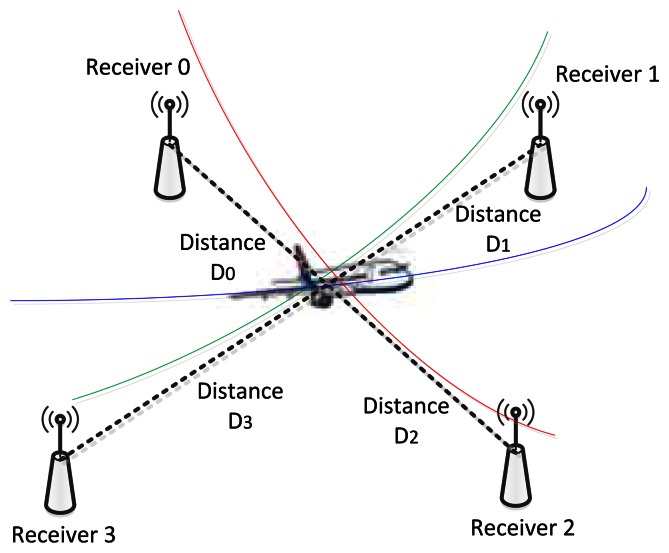
MLAT.

Thus, with four receivers, the three-dimensional coordinates (*x*, *y*, *z*) can be determined. If you receive altitude information from secondary surveillance radar, you can determine the aircraft position using only three receivers. Figure 1 shows how to measure aircraft position by MLAT. The aircraft position error in MLAT is known to be 3–6 m near an airport and 10–13 m at 16 km distance away from an airport [22].

### 2.5. WAM

MLAT techniques were developed by the military decades ago and have been successfully employed for airport surveillance more recently. Today, these same techniques are being used for larger areas to cover the en-route and approach phases of flight. Such systems are called WAM systems.

In WAM, the stations are spread much further apart, at distances of up to 100 km between each other. WAM installations in places such as Tasmania and the Czech Republic provide superior range over secondary radar, more accurate tracking, significantly lower costs, and significantly earlier operational readiness following contract award. Armenia has chosen WAM as a replacement for their existing secondary radar because cost and performance analyses have shown clear advantages for MLAT [23].

In addition, in the North Sea, between the UK and northern Europe and Scandinavia, small, lightweight, and low-powered MLAT units will be mounted on offshore drilling platforms to provide better performance down to the surface in locations where secondary radar would have been impractical. The aircraft position error of WAM is known to be 50 m at a distance of 192 km from an airport.

### 2.6. IMM Filter

In this study, we compare and analyze existing data with the IMM filter. Among the several useful multiple model filters that have been studied thus far, the IMM filter is known for having the computational capabilities of the first-order generalized pseudo-Bayesian (GPB1) estimator, but the performance of the second-order generalized pseudo-Bayesian (GPB2) estimator for GBAS, ADS-B, MLAT, WAM data [24,25]. The IMM filter is widely known and has been applied to a number of models since the early 1990s. Li *et al*. applied the IMM filter to the aircraft tracking problem [26]. One cycle of the IMM filter has a recursive structure that consists of four stages: interaction, prediction, update, and combination. Each time it is run, it combines the appropriate models from its model database and chooses the combination of aircraft motion models that best fits the position data. After choosing the model, it is adapted to the aircraft dynamics in order to generate the best representation of the aircraft motion model [27].

### 2.7. Sensor Fusion

Many theories are used in sensor fusion, but among them, centralized algorithms and distributed algorithms are the most widely used [28]. Centralized sensor fusion algorithms handle all measurements in one central filter. In this case, the computational load is high because all measurements should be calculated, necessitating high-performance hardware. Moreover, it is not easy to ensure robustness of the system to sensor failure or transient invalid input data. Despite these disadvantages, it has the advantage of affording the optimal solution [29].

Distributed sensor fusion algorithms do not handle all measurements in one central filter, but rather fuse all data in the main filter after processing each sensor data in a sub-filter specifically allocated to each sensor. In other words, they estimate the state variables and covariance through processing each radar data in a sub-filter. This estimate is delivered to the primary filter, and then, the overall optimum state variables are estimated.

## 3. Sensor Fusion with the IMM Filter

### 3.1. Applying the IMM Filter

In order to apply the IMM filter to each sensor, we created three models of aircraft movements: uniform motion, accelerated motion, and rotational motion. The first assumes a constant speed, the second, accelerated motion, and the third, following a circle at a constant speed.

A uniform motion model for an aircraft can be expressed as follows:

(4) $${\text{x}}_{\text{u}}(\text{k})=\left[\begin{array}{cccccc} 1 & 0 & T & 0 & 0 & 0\\ 0 & 1 & 0 & T & 0 & 0\\ 0 & 0 & 1 & 0 & 0 & 0\\ 0 & 0 & 0 & 1 & 0 & 0\\ 0 & 0 & 0 & 0 & 1 & 0\\ 0 & 0 & 0 & 0 & 0 & 1 \end{array}\right]{\text{x}}_{\text{u}}(\text{k}-1)+\left[\begin{array}{cc} \frac{1}{2}T^{2} & 0\\ 0 & \frac{1}{2}T^{2}\\ T & 0\\ 0 & T\\ 0 & 0\\ 0 & 0 \end{array}\right]\text{v}(\text{k}-1)$$

where *T* is the sampling time, the average of noise v(k) is 0, and white noise is assumed to have a Gaussian distribution. The state vector of uniform motion x_u_(k), which consists of position, velocity and acceleration, is as follows:

(5) $${\text{x}}_{\text{u}}(\text{k})={\left[\begin{array}{cccccc} x & y & \dot{x} & \dot{y} & \ddot{x} & \ddot{y} \end{array}\right]}^{t}$$

Accelerated motion of the aircraft can be expressed as follows:

(6) $${\text{x}}_{\text{a}}(\text{k})=\left[\begin{array}{cccccc} 1 & 0 & T & 0 & \frac{1}{2}T^{2} & 0\\ 0 & 1 & 0 & T & 0 & \frac{1}{2}T^{2}\\ 0 & 0 & 1 & 0 & T & 0\\ 0 & 0 & 0 & 1 & 0 & T\\ 0 & 0 & 0 & 0 & 1 & 0\\ 0 & 0 & 0 & 0 & 0 & 1 \end{array}\right]{\text{x}}_{\text{a}}(\text{k}-1)+\left[\begin{array}{cc} \frac{1}{2}T^{2} & 0\\ 0 & \frac{1}{2}T^{2}\\ T & 0\\ 0 & T\\ 1 & 0\\ 0 & 1 \end{array}\right]\text{v}(\text{k}-1)$$

The state vector of accelerated motion **x_a_(k)** is the same as in uniform motion:

(7) $${\text{x}}_{\text{a}}(\text{k})={\left[\begin{array}{cccccc} x & y & \dot{x} & \dot{y} & \ddot{x} & \ddot{y} \end{array}\right]}^{t}$$

Rotational motion has a constant angular velocity. Rotational motion of the aircraft can be expressed as follows:

(8) $${\text{x}}_{\text{r}}(\text{k})=\left[\begin{array}{ccccccc} 1 & \frac{\sin\omega T}{\omega } & 0 & -\frac{1-\cos\omega T}{\omega } & 0 & 0 & 0\\ 0 & \cos\omega T & 0 & -\sin\omega T & 0 & 0 & 0\\ 0 & \frac{1-\cos\omega T}{\omega } & 1 & \frac{\sin\omega T}{\omega } & 0 & 0 & 0\\ 0 & \sin\omega T & 0 & \cos\omega T & 0 & 0 & 0\\ 0 & 0 & 0 & 0 & 1 & 0 & 0\\ 0 & 0 & 0 & 0 & 0 & 1 & 0\\ 0 & 0 & 0 & 0 & 0 & 0 & 1 \end{array}\right]{\text{x}}_{\text{r}}(\text{k}-1)+\left[\begin{array}{cc} \frac{1}{2}T^{2} & 0\\ 0 & \frac{1}{2}T^{2}\\ T & 0\\ 0 & T\\ 0 & 0\\ 0 & 0\\ 0 & 0 \end{array}\right]\text{v}(\text{k}-1)$$

The state vector of rotational motion consists of position, velocity, acceleration and a turn rate as follows:

(9) $${\text{x}}_{\text{r}}(\text{k})={\left[\begin{array}{ccccccc} x & y & \dot{x} & \dot{y} & \ddot{x} & \ddot{y} & \omega \end{array}\right]}^{t}$$

The position of aircraft can be obtained from the GBAS, ADS-B, MLAT, and WAM sensors. The measurement model is as follows:

(10) $$\text{z}(\text{k})=\left[\begin{array}{ccccccc} 1 & 0 & 0 & 0 & 0 & 0 & 0\\ 0 & 1 & 0 & 0 & 0 & 0 & 0 \end{array}\right]{\text{x}}_{\text{u},\text{a},\text{r}}(\text{k})+\text{w}(\text{k})$$

where **w(k)** is the measurement noise and **x_u.a.r_(k)** means **x_u_(k)** or **x_a_(k)** or **x_r_(k)**. Through this model, the IMM filter can be applied to each sensor. The procedure and equations of the IMM filter are given in [16]. The Gaussian distribution is used for the sensor error, and the system model is linear. We also assume that Markov transition matrix *π* and initial mode probability *μ* are as follows:

(11) $$\pi =\left[\begin{array}{cc} 0.95 & 0.05\\ 0.1 & 0.9 \end{array}\right]$$

(12) $$\mu =\left[\begin{array}{c} 0.5\\ 0.5 \end{array}\right]$$

**Figure 2.**
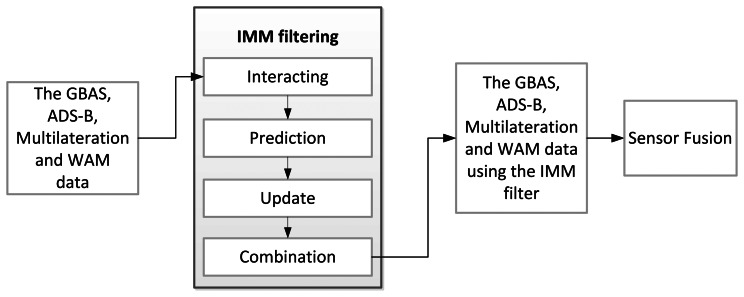
Flowchart for application of IMM filter.

Figure 2 shows the IMM filter procedure applied to each sensor.

### 3.2. Proposed Sensor Fusion Method

In order to fuse GBAS, ADS-B, MLAT and WAM, the sensor fusion method with the IMM filter is proposed. Assume that we obtain measurements from N sensors and the covariance and estimates for these N data are obtained with the IMM filter. The calculated estimates and covariances are sent to the main filter, which provides a final result.

Through the mixing process of the IMM filter, measurements from the N sensors are converted into estimates ${\tilde{x}}_{k-1}^{j}$ and ${\tilde{P}}_{k-1}^{j}$ at *t* = *k* −1. The prediction process applied to these values afford the estimates ${\bar{x}}_{k-1}^{j}$ and ${\bar{P}}_{k-1}^{j}$ at *t* = *k*. Further, through the process of updating, we obtain ${\hat{x}}_{k-1}^{j}$ and ${\hat{P}}_{k-1}^{j}$. Finally, through the process of combining each sensor data, we obtain the final estimates *x̂*_1_, *x̂*_2_ ⋯, *x̂_N_* and covariances *P_1_*, *P_2_* ⋯, *P_N_*. The resulting values are sent to the main filter. In the main filter, the final results are obtained using the following equations:

(13) $$\hat{x}=P\left[P_{1}^{-1}{\hat{x}}_{1}+P_{2}^{-1}{\hat{x}}_{2}+\cdots +P_{N}^{-1}{\hat{x}}_{N}\right]$$

(14) $$P={\left[P_{1}^{-1}+P_{2}^{-1}+\cdots +P_{N}^{-1}\right]}^{-1}$$

**Figure 3.**
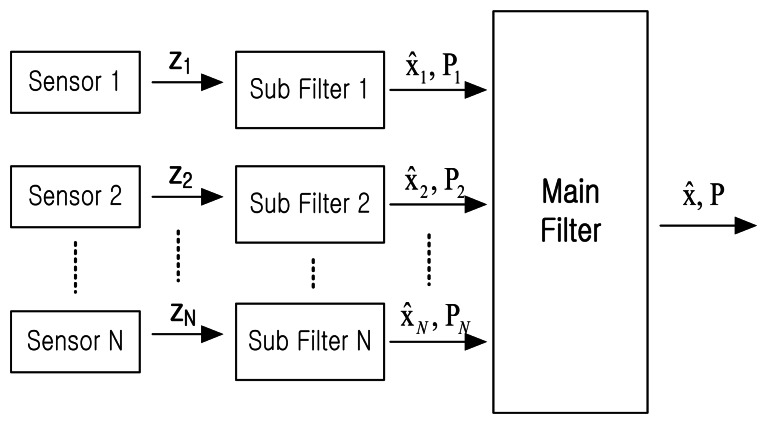
Proposed sensor fusion method with the IMM filter.

*x̂*_1_, *x̂*_2_ ⋯, *x̂_N_* comprise the final estimate calculated by the sub-filters; and *P*_1_, *P*_2_ ⋯, *P_N_* are the covariances calculated by the sub-filters. *x̂* and *P* are the final results obtained from the main filter. The proposed sensor fusion method is shown in Figure 3.

## 4. The Performance Evaluation

Performance evaluation of the proposed sensor fusion algorithm with the IMM filter was performed using MATLAB and all simulations were performed by Monte Carlo method 1,000 times.

### 4.1. Data Set

We used virtual sensor data. In addition, we set up and simulated three scenarios with targets at different distances from the airport, as shown in Table 1. We believe the proposed scenarios in this paper are perfectly practical and appropriate for air traffic control. First of all, there has not been any practical set of scenarios regarding aircraft movements reported in literature, at least to the very best of our knowledge. The presented scenarios (1, 2, and 3) reflect the fact that aircrafts near an airport undergo three motions—uniform motion, accelerated motion, and rotational motion—and that those away from the airport only undergo uniform motion. Also considering how GBAS and MLAT sensor can only be used near an airport, the proposed scenarios seem perfectly practical.

The virtual data is the true position plus random noise added on the basis of the accuracy of each sensor. We added sensor failure, sensor signal loss, and multipath interference for more realistic simulations. Sensor failure and sensor signal loss are assumed to correspond to a Poisson process with *λ* corresponding to the average arrival rate. If *Y* is the time between signal loss occurrences in the Poisson process, the probability that the time *Y* exceeds t seconds is equivalent to having no signal loss for t seconds. The probability is as follows:

(15) $$\begin{array}{ll} P\left[Y>t\right] & =P\left[\text{no signal loss in t seconds}\right]\\ & ={\left(1-P\right)}^{n}\\ & ={\left(1-\frac{\lambda t}{n}\right)}^{n}\\ & =e^{-\lambda t}\;asn\to \infty \end{array}$$

Therefore, *Y* is an exponential random variable with parameter *λ*. We assumed that the average arrival rate is 0.035 per second by based on of air-to-ground communication systems. According to this process, sensor failure and sensor signal loss are generated. The Gaussian distribution is used for the error model. The covariance of error for each sensor is 3 and the magnitude of each sensor is based on their error.

### 4.2. Scenario 1

**Figure 4.**
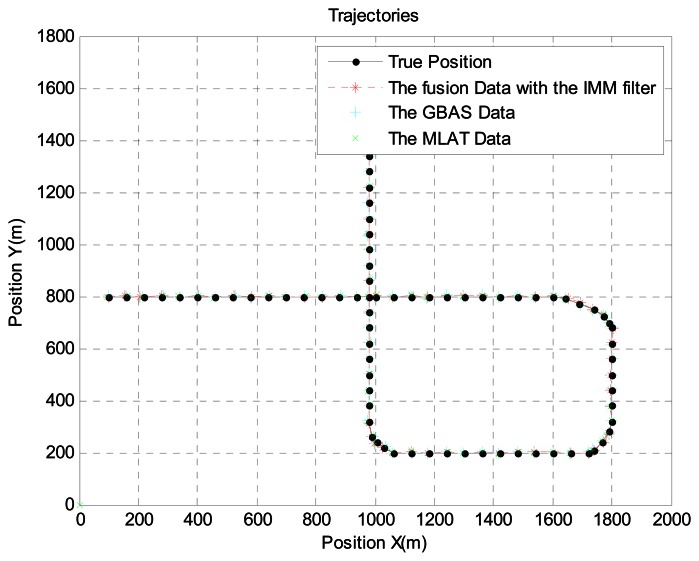
Aircraft trajectory near the airport.

**Figure 5.**
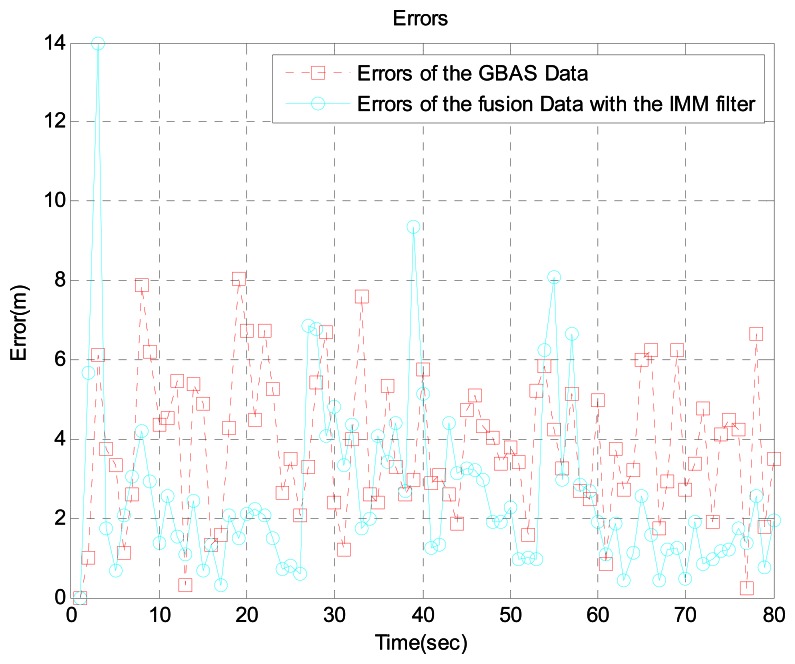
Errors between GBAS data and fusion data with the IMM filter near the airport.

**Figure 6.**
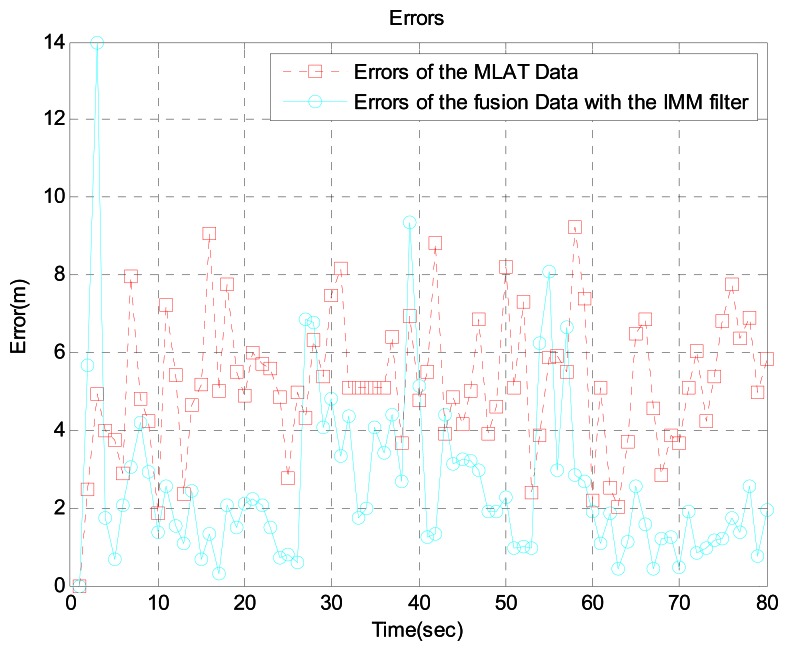
Errors between MLAT data and fusion data with the IMM filter near the airport.

In scenario 1, the target is near an airport. Because the speed is relatively low and there are many acceleration and turning movements, we generated the aircraft trajectory shown in Figure 4, which shows the aircraft's true position, GBAS data, MLAT data, and the sensor fusion data with the IMM filter. For a more realistic simulation, we added MLAT failure and MLAT signal loss using the Poisson process. Error usually increases near airports because of multipath interference. Therefore, we increased the error by 5 percent. Figure 5 shows the error between the GBAS data and the sensor fusion data with the IMM filter. Likewise, Figure 6 shows the error between the MLAT data and the sensor fusion data with the IMM filter. As shown in the figure, the sensor fusion data with the IMM filter has less error than the original GBAS and MLAT data.

### 4.3. Scenario 2

**Figure 7.**
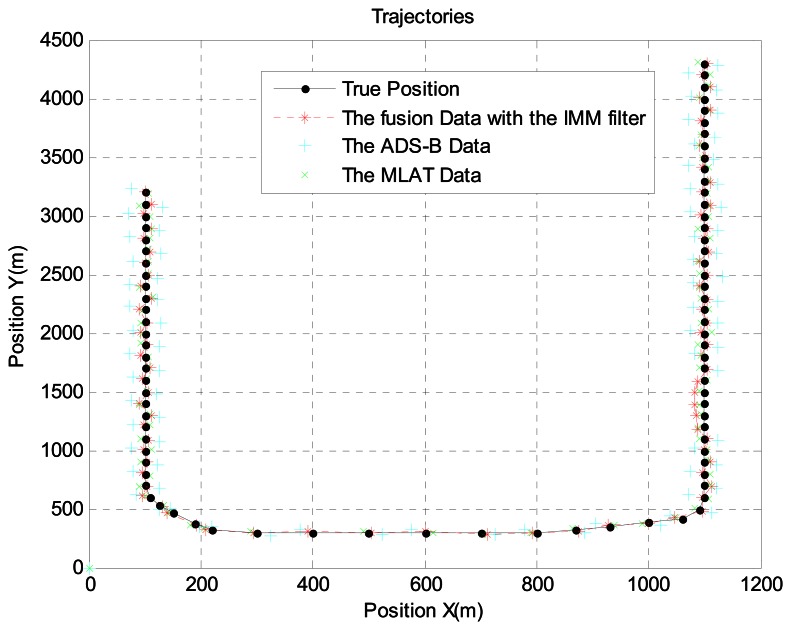
Aircraft trajectory at 16 km from the airport.

**Figure 8.**
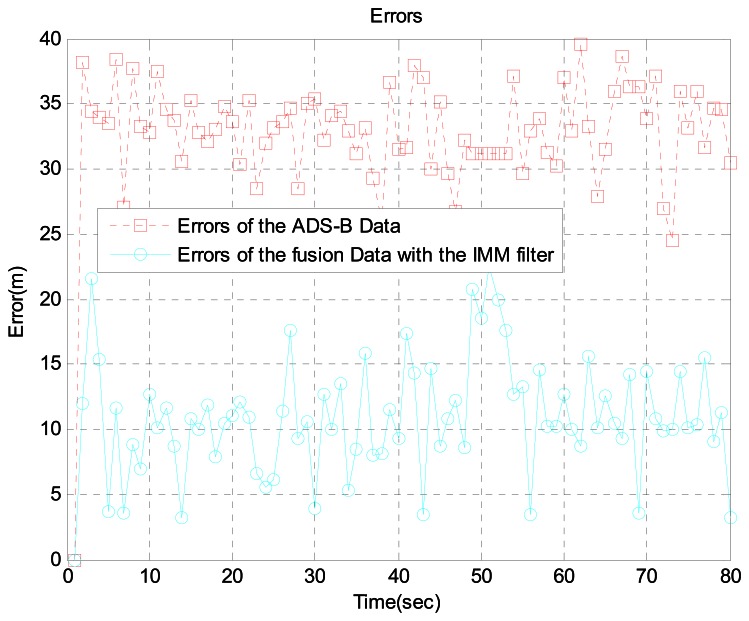
Errors between ADS-B data and fusion data with the IMM filter at 16 km from the airport.

**Figure 9.**
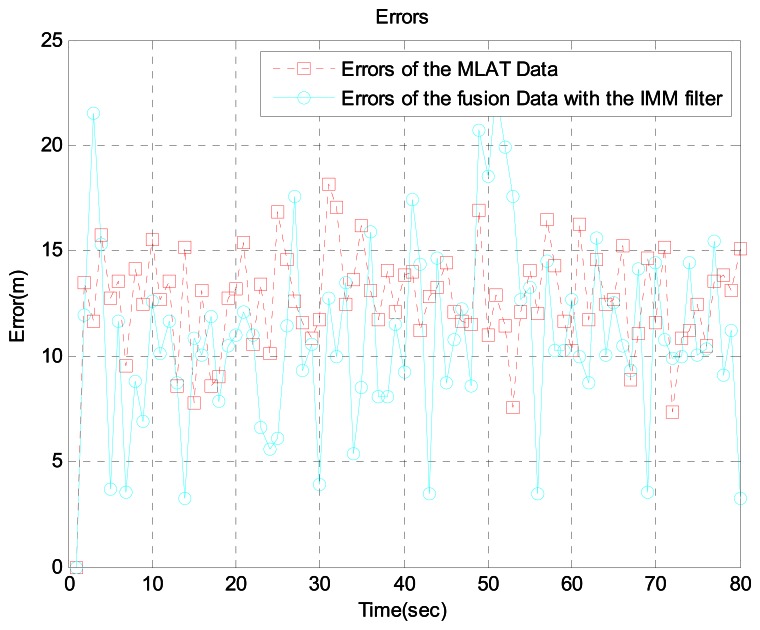
Errors between MLAT data and fusion data with the IMM filter at 16 km from the airport.

In scenario 2, the target is 16 km away from the airport. Because there are many acceleration and turning movements in this area, we generated the aircraft trajectory shown in Figure 7, which shows the aircraft's true position, ADS-B data, MLAT data, and sensor fusion data with the IMM filter. For a more realistic simulation, we added ADS-B failure and MLAT signal loss using the Poisson process. Figure 8 shows the error between ADS-B data and the sensor fusion data with the IMM filter. In addition, Figure 9 shows the error between the MLAT data and the sensor fusion data with the IMM filter. As shown in the figures, the sensor fusion data with the IMM filter has less error than the original ADS-B data and MLAT data.

### 4.4. Scenario 3

**Figure 10.**
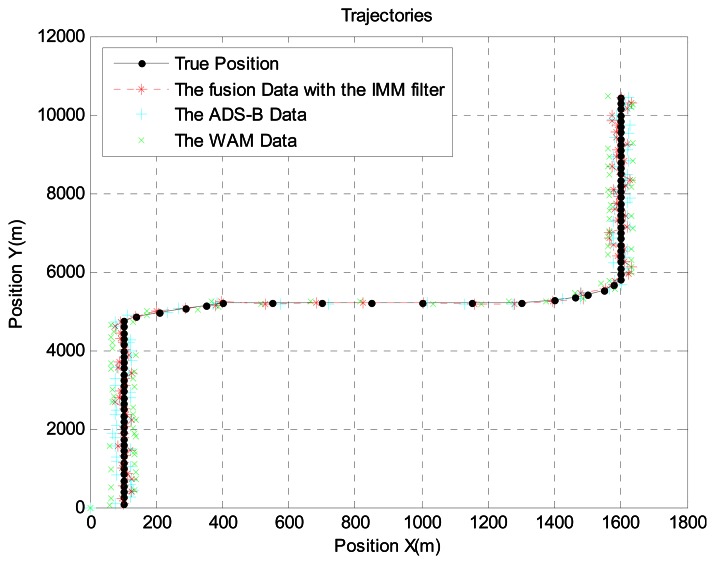
Aircraft trajectory at 192 km from the airport.

**Figure 11.**
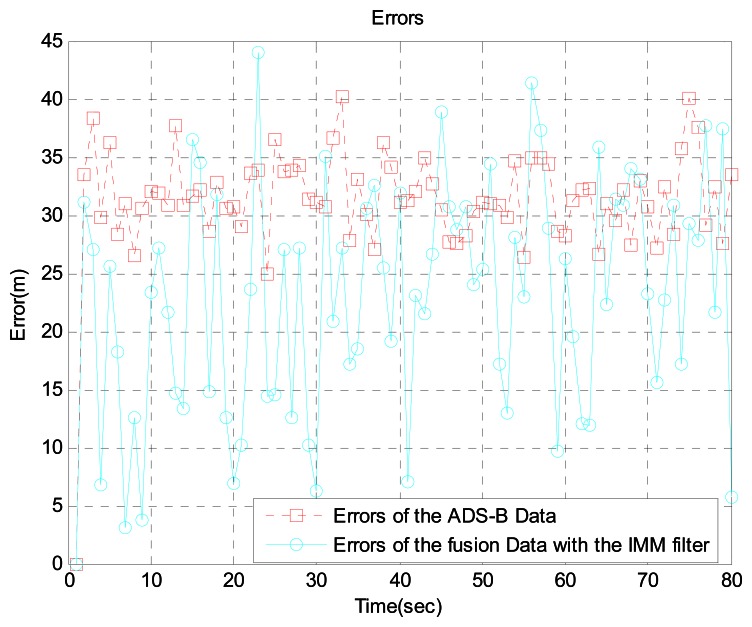
Errors between ADS-B data and fusion data with the IMM filter at 192 km from the airport.

In scenario 3, the target is 192 km from the airport. We generated the aircraft trajectory shown in Figure 10 because uniform motion is common, rather than acceleration and turning, at this distance. Figure 10 shows the aircraft's true position, ADS-B data, WAM data, and sensor fusion data obtained with the IMM filter. For more realistic simulations, we added WAM failure and ADS-B signal loss using the Poisson process. Figure 11 shows the error between the ADS-B data and sensor fusion data.

**Figure 12.**
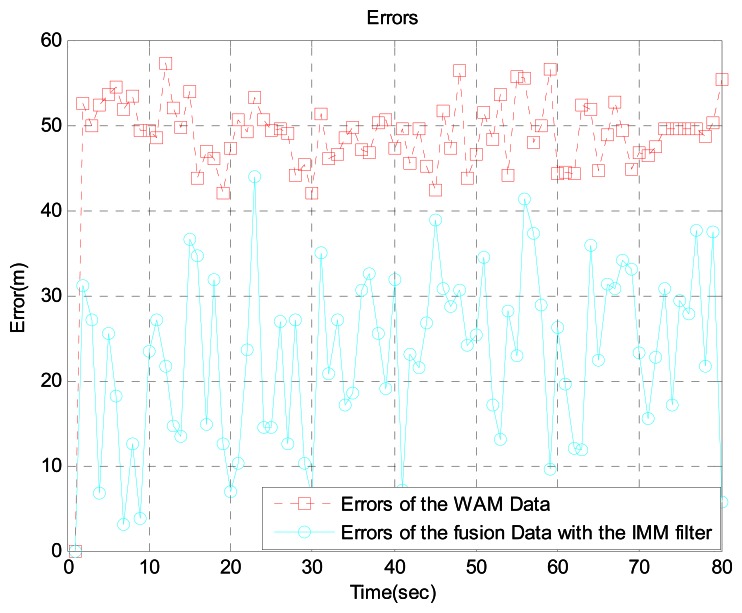
Errors between WAM data and fusion data with the IMM filter at 192 km from the airport.

Likewise, Figure 12 shows the error between the WAM data and sensor fusion data. As seen in the figures, the sensor fusion data has less error than the original ADS-B data and WAM data.

### 4.5. Results and Discussions

In scenarios 1, 2, and 3, our approach performed better than existing method. Table 2 summarizes the error in each sensor scenario. These errors are root mean square (RMS) values. Clearly, the proposed method achieves performance enhancement in all three scenarios up to 68.61%. GBAS and MLAT are more accurate than ADS-B and WAM, so the improvement of our approach is small. On the other hand, greater performance enhancement is observed in ADS-B and WAM.

On rare occasions, the proposed fusion method gives poorer estimates of predicted aircraft location when the motion model is changed or initialized. For example, we can find them at times 3 s, 39 s, and 55 s in Figure 5. During initialization or a change in the motion model, it is very difficult to decide which aircraft motion model suits best. In such cases, our method may yield poorer estimates. However, we do want to emphasize that such cases are very rare, rendering the overall average performance better than that of the existing methods.

## 5. Conclusion

In this paper, we presented an accurate method for determining aircraft position through sensor fusion with the IMM filter. The existing method is a simple fusion without IMM filter. Therefore, we applied the IMM filter to sensor fusion in order to improve the performance. The IMM filter is known for its good performance and is commonly used in radar target-tracking filters. We tested the method for aircraft moving with uniform motion, accelerated motion, and rotation. The proposed method affords more reliable aircraft positions than each other type of sensor. We set up and simulated three scenarios with various aircraft distances from the airport. The simulation results showed an average performance improvement of 40.93% compared with existing sensor data.

Future research will focus on utilizing the recently developed particle filter. On very rare occasions, the proposed fusion method gives poorer estimates of predicted aircraft location. We believe the particle filter can be used to solve this problem, and will be particularly useful as it can be applied to any state model and nonlinear systems. Moreover, we are already in the process of solving this problem by implementing the particle filter to our system. Currently, we are trying to modify the particle filter to reduce the calculation time.

Our discussion on sensors such as GBAS, ADS-B, MLAT, and WAM in the present paper is limited to simulation results as no airport except some in Australia and the U.S. are currently equipped with such sensors. Starting in 2015, we hope to be able to expand our scope to actual sensor data generated by airports that are scheduled to operate these sensors.

## Figures and Tables

**Table 1.** Simulation scenarios.

**Scenario**	**Distance from the Airport**	**Sensors Used**
Scenario 1	Near the airport	GBAS
		MLAT
Scenario 2	16 km from the airport	ADS-B
		MLAT
Scenario 3	192 km from the airport	ADS-B
		WAM

**Table 2.** Error of each sensor in each scenario.

	**GBAS (m)**	**ADS-B (m)**	**MLAT (m)**	**WAM (m)**	**Sensor Fusion (m)**	**Performance Improvement**	

						***vs.*GBAS/ADS-B**	***vs.*MLAT/WAM**
Scenario 1	3.84	-	5.22	-	2.68	30.20%	48.65%
Scenario 2	-	32.78	12.65	-	10.29	68.61%	18.65%
Scenario 3	-	31.30	-	48.60	22.95	26.68%	52.78%
